# Assessing the Value of Incorporating a Polygenic Risk Score with Nongenetic Factors for Predicting Breast Cancer Diagnosis in the UK Biobank

**DOI:** 10.1158/1055-9965.EPI-23-1432

**Published:** 2024-04-17

**Authors:** Jennifer A. Collister, Xiaonan Liu, Thomas J. Littlejohns, Jack Cuzick, Lei Clifton, David J. Hunter

**Affiliations:** 1 Nuffield Department of Population Health, University of Oxford, Oxford, United Kingdom.; 2 Wolfson Institute of Population Health, Queen Mary University of London, London, United Kingdom.; 3 Department of Epidemiology, Harvard TH Chan School of Public Health, Boston, Massachusetts.

## Abstract

**Background:**

Previous studies have demonstrated that incorporating a polygenic risk score (PRS) to existing risk prediction models for breast cancer improves model fit, but to determine its clinical utility the impact on risk categorization needs to be established. We add a PRS to two well-established models and quantify the difference in classification using the net reclassification improvement (NRI).

**Methods:**

We analyzed data from 126,490 post-menopausal women of “White British” ancestry, aged 40 to 69 years at baseline from the UK Biobank prospective cohort. The breast cancer outcome was derived from linked registry data and hospital records. We combined a PRS for breast cancer with 10-year risk scores from the Tyrer–Cuzick and Gail models, and compared these to the risk scores from the models using phenotypic variables alone. We report metrics of discrimination and classification, and consider the importance of the risk threshold selected.

**Results:**

The Harrell's C statistic of the 10-year risk from the Tyrer–Cuzick and Gail models was 0.57 and 0.54, respectively, increasing to 0.67 when the PRS was included. Inclusion of the PRS gave a positive NRI for cases in both models [0.080 (95% confidence interval (CI), 0.053–0.104) and 0.051 (95% CI, 0.030–0.073), respectively], with negligible impact on controls.

**Conclusions:**

The addition of a PRS for breast cancer to the well-established Tyrer–Cuzick and Gail models provides a substantial improvement in the prediction accuracy and risk stratification.

**Impact:**

These findings could have important implications for the ongoing discussion about the value of PRS in risk prediction models and screening.

## Introduction

Breast cancer is the most common cancer worldwide among women (1). There are a variety of breast cancer risk models available to predict risk of cancer, ranging from hormonal/environmental models, such as the Gail and Rosner–Colditz models (2–4), that typically incorporate hormonal risk factors, lifestyle factors and a limited family history. Other models estimate major gene carrier probability, such as the Manchester Scoring System (5) using a more detailed family history assessment or pedigree to determine the probability of a woman carrying a high-risk mutation (e.g., *BRCA1*, *BRCA2*) that might confer a predisposition to breast cancer. Some models incorporate both aspects; for example, the BOADICEA model (6, 7) and Tyrer–Cuzick model (8, 9).

In addition to the high-penetrance risk variants in a few key genes that dramatically increase breast cancer risk, recent genome-wide association studies (GWAS) have identified hundreds of common genetic variants with smaller effects on breast cancer risk (10, 11). These can be combined into polygenic risk scores (PRS) to provide a summary measure of the polygenic predisposition to breast cancer. Although some risk models have incorporated genetic information in the form of PRS or known pathogenic mutations in high-risk variants (in the woman or her family), these models are more likely to be used among women already known to be at high risk (12), as this polygenic information is not yet widely available in the general population.

With the increasing availability of, and interest in, genetic data, in particular PRS, further work is needed to explore to what extent PRS can contribute to general population risk stratification over and above the existing risk models. Supplementing clinical risk scores with PRS has been shown to improve their predictive performance (13–18), but most work in this area has been limited to showing improvements in discrimination. A better understanding of the change in risk categorization of individuals is necessary before this could be applied in the general population.

In this study, we aim to address this gap using two well-established and freely available breast cancer risk models, the Tyrer–Cuzick and Gail models, in a large population cohort. We compute the 10-year risk scores from both models, and compare the performance and risk classification of these scores with and without a breast cancer PRS included. We assess these in the UK Biobank, a cohort for whom the self-reported risk factor information available is typical of what can be typically elicited in the general population, and use a multiethnic polygenic risk score for breast cancer (PRS_BC_) recently developed by Genomics PLC (medRxiv 2022.06.16.22276246).

## Materials and Methods

### Study population

The UK Biobank (UKB; RRID:SCR_012815) is an ongoing large-scale population-based prospective cohort study of approximately 500,000 individuals aged 40 to 69 years recruited across the United Kingdom between March 2006 and October 2010 (19, 20). During baseline assessment at recruitment, data were collected in person via touchscreen questionnaires, a nurse-led verbal interview, physical examinations, and from a range of biological samples.

Our study population consists of postmenopausal female participants who had nonmissing core variables [including PRS and valid self-reported data on hormone replacement treatment (HRT) use] at recruitment. We restricted to postmenopausal women because premenopausal and postmenopausal breast cancers have different characteristics and risk patterns, and the majority of participants are postmenopausal (∼60%) due to the age range recruited. Because there were so few individuals of non-European ancestry in the UK Biobank, we restricted our study population to the genetically “White British” population (UKB Field 22006)—individuals who self-reported as White British and who had very similar ancestral backgrounds according to principal components analysis with k-means clustering (21).

We excluded participants with a prevalent breast cancer diagnosis, and those who had previously been diagnosed with carcinoma-in-situ of the breast based on two sources of data: (i) cancer registries, and (ii) hospital inpatient records using International Classification of Diseases codes (ICD-9: 174*, 2330 and ICD-10: C50*, D05*), with the date of diagnosis preceding or on the date of baseline assessment. We also excluded participants who had undergone mastectomy prior to baseline based on hospital inpatient records using OPCS Classification of Intervention and Procedures version 4 codes (OPCS4: B27*, B28.1, B28.2, B28.3, B28.4, B28.6, B41*), as they are at reduced risk of breast cancer.

### Breast cancer outcome

Incident breast cancer (ICD-9: 174* and ICD-10: C50*) was derived from cohort-wide linked medical data. The main source was cancer registry data (available until 31 December, 2020 in England, 31 December, 2016 in Wales, and 30 November, 2021 in Scotland), supplemented with hospital inpatient records and death registries to capture longer total follow-up. Total follow-up time was calculated as the number of years from baseline assessment until date of incident breast cancer, date of death from other causes, date of loss to follow-up, date of mastectomy or last date of medical record availability in UK Biobank (31 October, 2022 in England, 31 August, 2022 in Scotland, and 31 May, 2022 in Wales), whichever came first.

### Breast cancer PRS

We used the PRS for breast cancer developed on external multi-ethnic GWAS data by Genomics PLC, described as having “favorable results” relative to comparator scores, that has been made available in the UK Biobank (UK Biobank Field ID 26220) for participants who met their quality control (medRxiv 2022.06.16.22276246). The PRS (hereafter referred to as PRS_BC_) was calculated as the sum of the per-variant effect size multiplied by allele dosage, followed by centering and variance-standardization by ancestry.

### Imputation of HRT use

The Tyrer–Cuzick model expects HRT usage status to be categorised into: Never, Previous user (more than 5 years ago), Previous user (less than 5 years ago) or Current user. There is no “unknown” or obvious default value in the model specification. The questions asked in the UK Biobank touchscreen questionnaire include “Ever used hormone-replacement therapy” (Field ID 2814) and “Age last used hormone-replacement therapy” (Field ID 3546), to which women could respond that they were current users.

Not all women reporting HRT usage could remember the age at which they last used HRT (answering “Do not know”), so we could not determine the previous usage categorization (more or less than 5 years) for these women. We therefore imputed this by determining the proportion of women who reported having stopped using HRT more than 5 years ago within each year of age, and randomly allocating the previous usage categorization of the women with missing information according to the reported proportions within each age category.

### Calculation of risk scores

The Tyrer–Cuzick (or IBIS) model includes the following lifestyle and reproductive factors associated with breast cancer risk: age, height, weight, age at first menstrual period, parity, age at first childbirth, menopausal status, age at menopause, use of HRT, history of ovarian cancer and atypical hyperplasia of the breast, family history, mammographic density and PRS. Supplementary Table S1 contains a full description of how these variables were derived in this study. Mammographic density was unavailable, and for consistency of methods between models, PRS_BC_ was not directly included (see the section “Sensitivity analyses” for details). For most factors, the Tyrer–Cuzick model has default values to use for missing data so we have not imputed these values, with the exception of HRT use duration which we imputed as described above. We then calculated 10-year risks using the IBIS breast cancer risk evaluation tool v8.0b (https://ems-trials.org/riskevaluator/).

The Gail (or BCRA) model includes age, age at first menstrual period, age at first live birth, number of affected first-degree relatives, number of benign breast biopsies, and atypical hyperplasia diagnoses. See Supplementary Table S2 for details of the UK Biobank Data Fields used. We calculated 10-year risks using the BCRA R package (v2.1.2).

### Statistical analysis

#### Descriptive analyses

Age-specific rates for each 5-year interval between 55 and 80 years were calculated as the number of cases identified in that interval divided by the number of person-years at risk. Age-standardized rates were calculated by direct standardization using the 2013 European Standard Population ages 55 to 80 years (https://ec.europa.eu/eurostat/web/products-manuals-and-guidelines/-/KS-RA-13–028).

Breast cancer incidence rates in the whole UKB population were calculated including prevalent cases, to align more closely with the rates found in national registry data. Individuals were considered at risk from age 55 until diagnosis of cancer, or censoring. Rates were also calculated in the analysis population of postmenopausal “White British” women without a prior history of breast cancer, carcinoma *in situ* or risk reduction mastectomy. Individuals were considered at risk from entry to the UKB study until diagnosis of cancer, or censoring.

As a descriptive analysis, and for comparison with the rates used in the derivation of the models, we computed age-adjusted and multivariable hazard ratios associated with the individual variables included in each model.

#### Risk scores with PRS_BC_

To incorporate PRS_BC_ into the models, we randomly split the analysis population into 80% training and 20% test datasets. For each model, we constructed a Cox proportional hazards model for time to breast cancer outcome in the training data, including PRS_BC_ and the 10-year risk from that model as covariates and adjusting for genetic array and the first 4 principal components of genetic ancestry (22).

These Cox models were then used to predict 10-year risks from Tyrer-Cuzick with PRS_BC_, and Gail with PRS_BC_ in the test data. The proportional hazards assumption was assessed visually with Schoenfeld residuals, using the survminer package.

#### Calibration of risk scores

To assess how closely the predicted 10-year breast cancer risks agreed with observed 10-year breast cancer risks in the UK Biobank, we plotted the observed proportion of women with breast cancer within 10 years of follow-up against the mean predicted 10-year risk of breast cancer, by deciles of predicted risk using the whole study population. To provide a more fair comparison with the predicted 10-year risks from the Tyrer–Cuzick and Gail models with PRS_BC_, that were predicted from Cox models developed in the training data, we recalibrated the Tyrer–Cuzick and Gail models using the calibration slope and intercept calculated in the 80% training set.

#### Model comparison and performance assessment

We present model comparison and performance assessment in the test data only, using the calibrated 10-year risk scores for both models.

Model discrimination was assessed in the 20% test data using Harrell's C index, an extension of the area under a receiver-operator characteristic curve to censored survival data. We compared the risks from the Tyrer–Cuzick and Gail models to our predicted risks with PRS_BC_ added to the models, and also to PRS_BC_ alone.

We compared the risk classification of women with and without PRS_BC_ using the net reclassification improvement (NRI; ref. 23), calculated for time-to-event data using the nricens package (RRID:SCR_025138). Because we can expect the inclusion of a PRS to identify more women at higher risk simply because we have incorporated an additional risk factor, a comparison based on a fixed risk threshold does not seem equitable, so we considered instead the classification of a fixed proportion of women. To do this, we determined the proportion of women considered to be at “moderate” or higher risk by each model using a 10-year breast cancer risk threshold of 5% or greater, which is compatible with previous work with the Tyrer–Cuzick model (24). For each model we then found the risk threshold at which the same proportion of women were considered to be at high risk according to the 10-year risk scores with PRS included.

Finally, we used decision curve analysis to provide a visualization of the net benefit of the risk scores across a range of risk thresholds (25). Considering a simplified scenario in which risk models could be used to identify women eligible for screening, or some other intervention, we can approximate the net benefit as a result of their use. The net benefit summarizes potential benefits and harms for a given risk threshold—the higher the net benefit, the better the model (26).

All statistical tests were two-tailed, at a 5% significance level. All analyses were performed using R Statistical Software (version 4.3.1; RRID:SCR_001905).

#### Sensitivity analyses

Our main analysis preserves a fixed proportion of women classified as high risk, to account for a healthcare system in which there may be a fixed budget for interventions among high-risk individuals. However, to explore the scenario where there is potential to increase the capacity for interventions when additional women are identified at high risk, we additionally calculated the NRI between risk scores with and without PRS_BC_ when a fixed 5% 10-year risk threshold was used for both.

Although the Gail model does not include a PRS, the latest version (v8) of the Tyrer–Cuzick model has the option to include a PRS directly, requiring that the PRS be entered in the form of a log OR for the breast cancer outcome, where the average risk is calibrated to the population. Using the correction proposed by Pharaoh and colleagues (27), we included PRS_BC_ directly in the Tyrer–Cuzick model, and compared the risk scores obtained in this way to those from the Cox model.

Finally, we investigated the impact of the choice of PRS, repeating the main analyses using a 313-variant PRS for breast cancer developed in individuals of European ancestry by the BCAC in place of the Genomics PLC score (10). This score (hereafter referred to as PRS_313_) is available in the Polygenic Score Catalog as PGS000004 (28).

### Ethics approval and consent to participate

The UK Biobank received ethical approval from the National Health Service (NHS) North West Centre for Research Ethics Committee (ref: 11/NW/0382). All participants gave written informed consent before enrolment in the study, which was conducted in accordance with the principles of the Declaration of Helsinki.

### Data availability

This research has been conducted using the UK Biobank Resource under Application Number 33952. Requests to access the data should be made via application directly to the UK Biobank, https://www.ukbiobank.ac.uk.

The code used for analyses are available at https://github.com/2cjenn/PRSforBrCa.

## Results

Of 502,413 participants in UKB, a total of 126,490 participants remained after applying the exclusion criteria, (Supplementary Fig. S1). Of these, 5,601 (4.4%) developed breast cancer over a median of 13.6 (IQR = 12.8–14.3) years of total available follow-up, and 4197 (3.3%) developed breast cancer within 10 years of follow-up.

### Baseline characteristics

The characteristics of our study population are presented in Supplementary Tables S3A–S3G. To summarize, participants in each quintile of PRS_BC_ were comparable across almost all sociodemographic and reproductive risk factors. Women in higher quintiles of PRS_BC_ were more likely to have had a prior breast biopsy, and to have a first-degree family history of breast cancer than women in lower quintiles.

Considering the age range 55 to 85 years, where women are likely to be postmenopausal, we observed age-standardized breast cancer incidence of 344.8 cases per 100,000 person years of follow-up among all women in the UK Biobank, which fell to 334.8 when restricting to our analysis population of postmenopausal White British women with complete data and no history of breast cancer or risk-reducing mastectomies. This is comparable with the 362.6 observed in the UK by the Office for National Statistics (ONS) in 2013 for the age range 55–85 years. Age specific incidence rates in UK Biobank were similar to those observed in ONS data (Supplementary Fig. S2).

**Figure 1.**
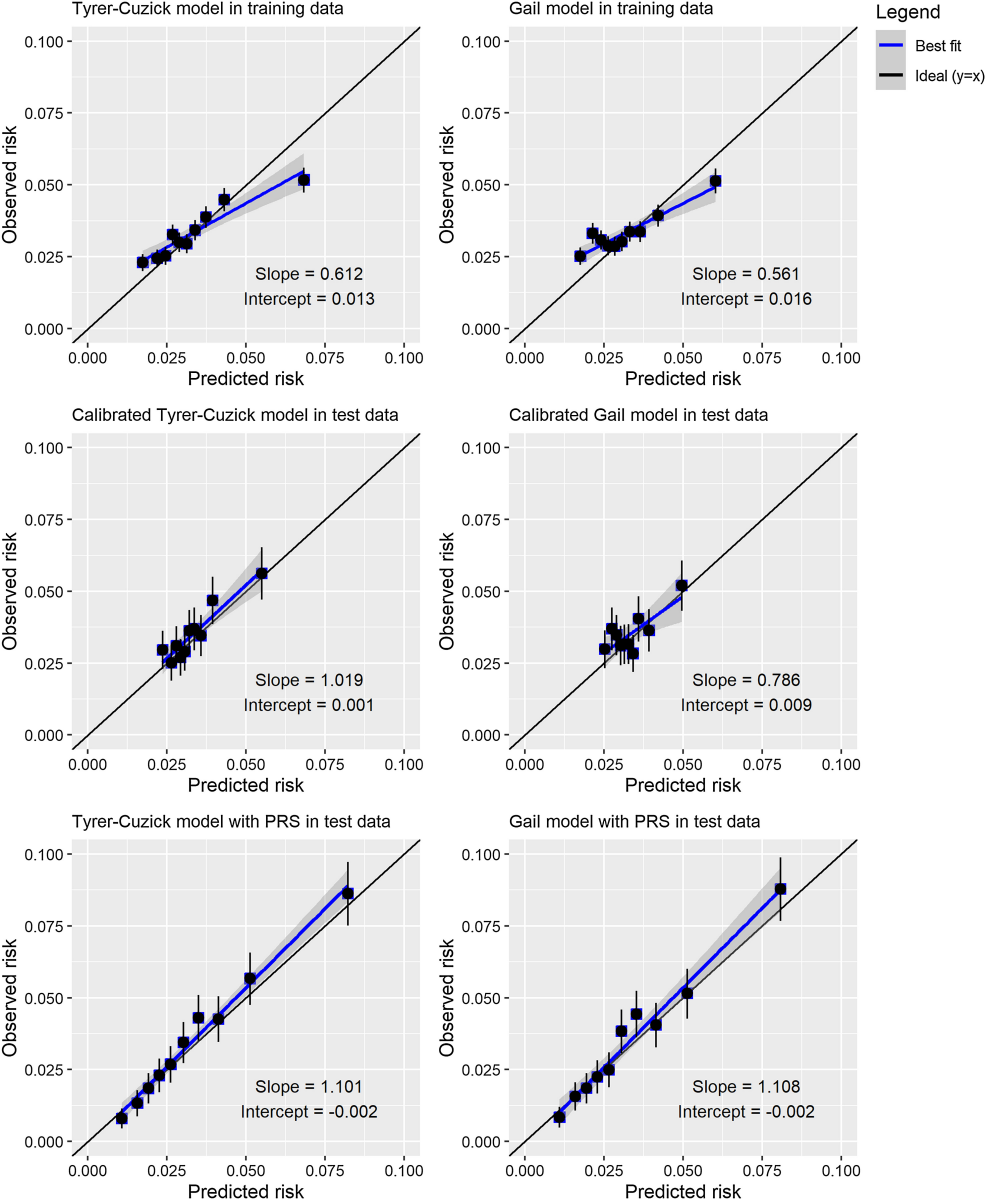
Calibration plots of 10-year observed versus predicted risk by risk decile, for the Tyrer–Cuzick and Gail models, plotting the observed proportion of women with breast cancer by 10 years of follow-up against the predicted 10-year risk from the models. Top row, predicted risks in training dataset from Tyrer–Cuzick and Gail models. Middle row, predicted risks in test dataset from Tyrer–Cuzick and Gail models, calibrated using the training dataset. Bottom row, predicted risks in test dataset from Cox model containing Tyrer–Cuzick or Gail model and PRS_BC_, developed in training dataset.

Both the Tyrer–Cuzick and Gail models overestimated the 10-year risk of breast cancer in the top risk decile of the training data and slightly underestimated risk in the bottom decile (Fig. 1). When constructing a Cox model with the original model 10-year risk score and PRS_BC_ in the training data, adjusting for genetic array and the first four principal components of ancestry, the Tyrer–Cuzick model appeared slightly more predictive than the Gail, with a HR per SD of 1.19 (95% CI, 1.16–1.22) compared with 1.15 (95% CI, 1.12–1.17).

Schoenfeld residual plots for both models are shown in Supplementary Figures S3 and S4. Age-adjusted and multivariable HRs calculated in our analysis population for the individual variables included in both models are presented in Supplementary Tables S4 and S5, and are broadly similar to the rates used in the derivation of the models (2, 29).

### Model performance

**Figure 2.**
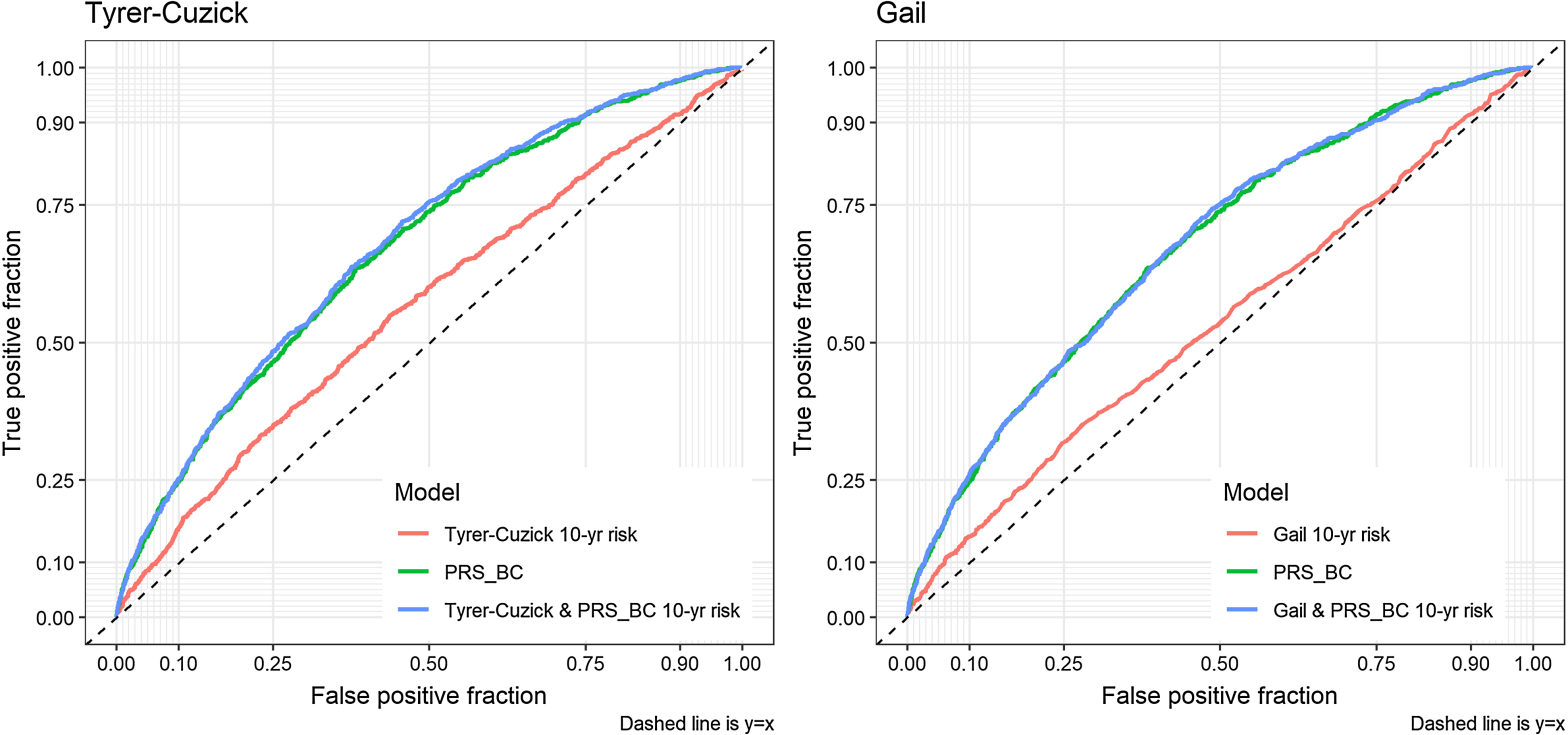
Time-dependent ROC plots at 10 years of follow up for both the Tyrer–Cuzick (left) and Gail (right) models, with and without PRS_BC_, as well as PRS_BC_ alone in test data (*N* = 25,369).

Comparing the 10-year risk scores from the calibrated original models to the scores obtained from 10-year risk predictions from these Cox models, we find that both breast cancer models are substantially improved by inclusion of PRS_BC_ (Fig. 2) with Harrell's C statistic increasing from 0.57 to 0.67 for the Tyrer–Cuzick, and 0.54 to 0.67 for the Gail model (Table 1) Notably, the ROC curve for each model with PRS_BC_ is almost indistinguishable from the curve for PRS_BC_ alone.

**Table 1.** Model performance with and without PRS_BC_ in test data (*N* = 25,369).

		NRI^a^		
Model	Harrell's C (95% CI)	Overall^b^	Cases (*N* = 877)	Controls (*N* = 23,355)
Tyrer–Cuzick				
Model only	0.57 (0.55–0.58)	0.083 (0.055–0.108)	0.080 (0.053–0.104)	0.003 (-0.001–0.007)
Model with PRS_BC_	0.67 (0.66–0.69)			
Gail				
Model only	0.54 (0.52–0.56)	0.053 (0.032–0.074)	0.051 (0.030–0.073)	0.002 (-0.001–0.004)
Model with PRS_BC_	0.67 (0.65–0.68)			

^a^NRI at 10 years of follow-up for 10-year risks from model with PRS_BC_ compared to calibrated model, with a fixed proportion of women classified as high risk (6.47% for Tyrer–Cuzick and 2.40% for Gail).

^b^Overall NRI = Case NRI + Control NRI, where cases are defined as individuals diagnosed with breast cancer within 10 years and controls are defined as individuals who were still at risk of breast cancer by 10 years of follow-up.

When considering the change in classification of individuals compared to the original models in the test data, we observed that inclusion of PRS_BC_ in both models improves categorization of cases (NRI_case_ = 0.080 for Tyrer–Cyzick and 0.051 for Gail), but has very little impact on the categorization of controls (NRI_control_ < 0.01 for both models). Note that breast cancer is a relatively rare outcome, so the overall population impact of the NRI_case_ will be small compared with the NRI_control_.

In the Tyrer–Cuzick model, inclusion of the PRS_BC_ led to 13% of future cases being reclassified into a higher risk category and 4% of controls moving down a category (Table 2). The picture was similar for the Gail model, with 8% of future cases being reclassified from “population” to “moderate” risk, and 2% of controls moving into a lower risk category (Table 3).

**Table 2.** Breakdown of case and control classification in the test data (*N* = 25,369) using 10-year risks from the Tyrer-Cuzick model with and without PRS_BC_, with a fixed proportion (6.47%) of women considered at “moderate risk”.

	Tyrer-Cuzick + PRS_BC_					Tyrer-Cuzick +PRS_BC_			
Cases		≤6.78%	>6.78%	Sum	Controls		≤6.78%	>6.78%	Sum
Tyrer–Cuzick	≤5%	678 (77.31%)	111 (12.66%)	789	Tyrer–Cuzick	≤5%	20,932 (89.63%)	943 (4.04%)	21,875
	>5%	41 (4.68%)	47 (5.36%)	88		>5%	1,018 (4.36%)	462 (1.98%)	1,480
	Sum	719	158	877		Sum	21,950	1,405	23,355

Note: Cases defined as individuals diagnosed with breast cancer within 10 years. Controls defined as individuals who were still at risk of breast cancer by 10 years of follow-up. Individuals censored before 10 years are not displayed.

**Table 3.** Breakdown of case and control classification in the test data (*N* = 25,369) using 10-year risks from the Gail model with and without PRS_BC_, with a fixed proportion (2.88%) of women considered at “moderate risk”.

	Gail + PRS_BC_					Gail + PRS_BC_			
Cases		≤8.40%	>8.40%	Sum	Controls		≤8.40%	>8.40%	Sum
Gail	≤5%	766 (87.34%)	70 (7.98%)	836	Gail	≤5%	22,228 (95.17%)	479 (2.05%)	22,707
	>5%	26 (2.96%)	15 (1.71%)	41		>5%	524 (2.24%)	124 (0.53%)	648
	Sum	792	85	877		Sum	22,752	603	23,355

Note: Cases defined as individuals diagnosed with breast cancer within 10 years. Controls defined as individuals who were still at risk of breast cancer by 10 years of follow-up. Individuals censored before 10 years are not displayed.

**Figure 3.**
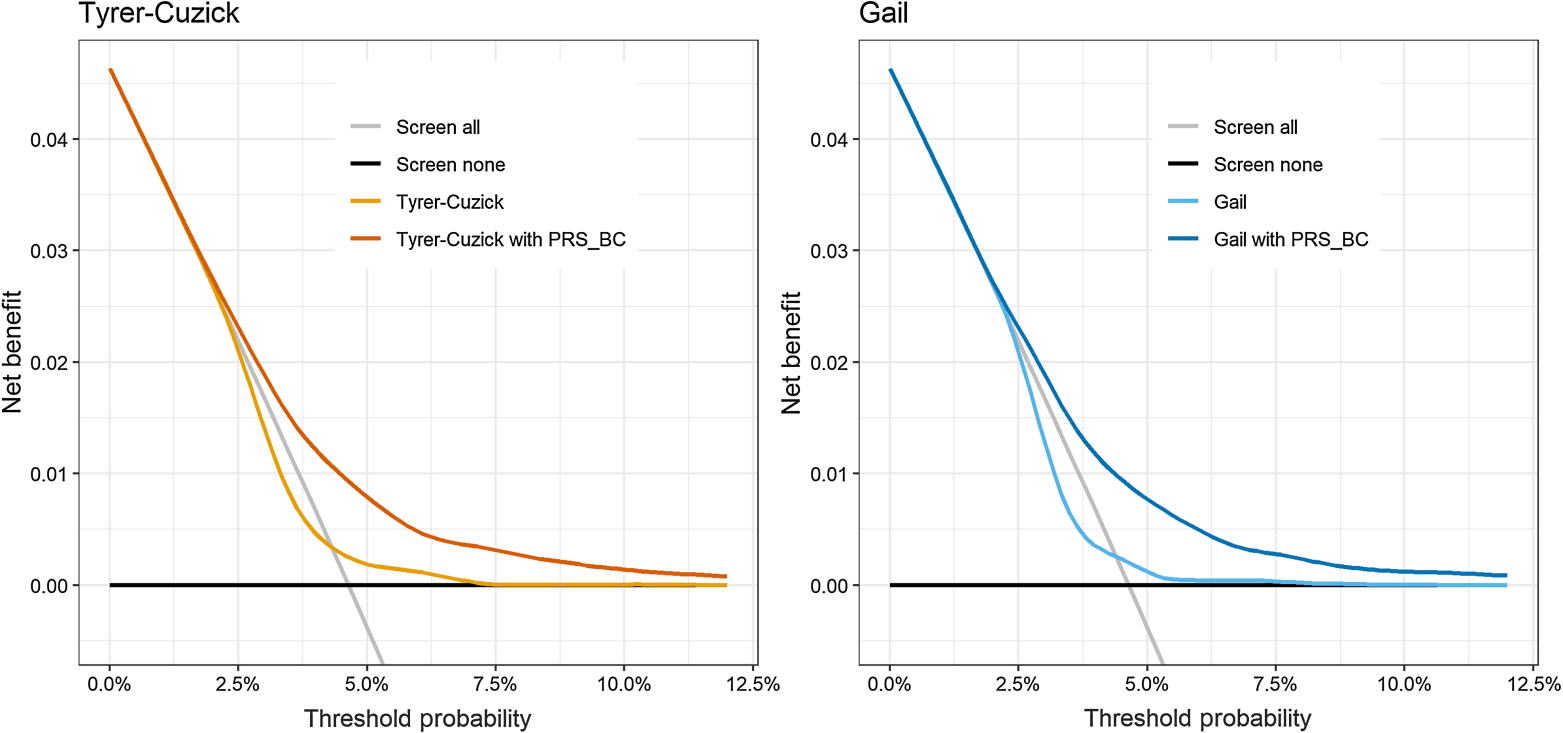
Decision curve analysis for Tyrer–Cuzick and Gail 10-year risk scores with and without PRS_BC_ in test data (*N* = 25,359). Plots show net benefit calculated in full available follow-up time. The gray line indicates net benefit if all women were screened, and the black line indicates net benefit if no women were screened.

Decision curve analysis (Fig. 3) illustrates that, over a range of possible threshold values of breast cancer risk, the models with PRS_BC_ give greater net benefit than without PRS_BC_. The threshold probability can be interpreted as the 10-year breast cancer risk at which the expected benefit of intervention is considered equal to the expected benefit of avoiding the intervention, and incorporates assumptions about the potential harm of false positives (e.g. anxiety and unnecessary treatment) versus the consequences of false negatives (not having treatment which would have been of benefit).

### Sensitivity analyses

When considering a fixed threshold of 5% 10-year risk for categorization of women both the Tyrer–Cuzick and Gail models with PRS_BC_ included identified around 34% of breast cancer cases as “moderate risk” compared with 10% categorized by the Tyrer–Cuzick model alone, and less than 5% by the Gail model (Supplementary Tables S6 and S7). However, both models also classified more noncase individuals as “moderate risk”. Thus, although the NRI_case_ is higher under this approach, the NRI_control_ is lower and this effect is magnified by the relatively few cases, meaning misclassification of unaffected women has a greater overall impact on the population.

This can be visualized in the density plots of 10-year risk scores from both models (Supplementary Fig. S5), where inclusion of PRS_BC_ is shown to “spread” the distribution of the risk score such that more individuals exceed the 5% threshold among both breast cancer cases and unaffected individuals.

When PRS_BC_ is incorporated directly into the Tyrer–Cuzick model, using the approach implemented in v8 of the model, the discrimination and NRI_case_ of the resulting 10-year risk score are virtually indistinguishable from those predicted from the Cox model containing the Tyrer–Cuzick 10-year risks and PRS_BC_ (Supplementary Table S8).

To consider the impact of the choice of PRS, we constructed 10-year risk scores from both models using PRS_313_, and evaluated their performance in the test data (Supplementary Table S9). The risk scores with PRS_313_ included have a slightly lower Harrell's C than those with PRS_BC_ (0.65 compared with 0.67 for the Tyrer–Cuzick, and 0.64 compared with 0.67 for the Gail model).

## Discussion

These findings may have implications for the potential future use of PRS in breast cancer screening recommendations. We have provided additional evidence that inclusion of a PRS improves the risk stratification of models containing conventional risk factors. We have also explored the impact that adding a PRS can have to the classification of individuals by such a score, which is of interest if a score currently in use were to be updated with the addition of a PRS and could have ramifications for screening recommendations and clinical guidance. Finally, under the assumption that screening would successfully detect cancers, our decision curve analysis indicated that across all thresholds considered, the net benefit of screening women according to their risk predicted from the models with PRS_BC_ was greater than that of screening all women indiscriminately, indicating inclusion of PRS_BC_ in such models could have benefit in determining women eligible for screening.

Our results are consistent with other work considering the value of PRS in breast cancer risk models (16, 30). In a large meta-analysis, Hurson and colleagues obtained an AUC of 0.64 from the iCARE model with PRS_313_ and projected that an improved PRS in combination with risk factors could attain an AUC of approximately 0.71 (31). Our analysis using the more recently developed Genomics plc PRS yields a Harrell's C of 0.67, suggesting there is still potential for improvements in the PRS. More recently Spaeth and colleagues considered the Gail model within the UK Biobank population, and compared it with BRISK, a newer risk model including a PRS (32). However, we have further shown that inclusion of a PRS can improve risk categorization of existing models that are currently used in practice, without requiring the development of new models.

The decision of risk threshold to use with a risk prediction model is a complex one, and can vary depending on the intended application; indeed it may often be desirable to consider model performance at a range of thresholds (33) or to consider age-dependent risk thresholds (34). When using a fixed threshold of absolute risk, it has been previously observed that combining breast cancer risk models with a PRS results in more women being identified as high risk (18, 35). Instead of comparing categorization by a fixed risk threshold, we evaluated whether inclusion of the PRS improves classification of individuals when the proportion of women considered to be at high risk is fixed, and found that it improved categorization of cases, with little impact on the categorization of controls.

A strength of this study is the large sample size and detailed data collection available from the UK Biobank, including genotyping array data and longitudinal linkage to electronic medical records over a long follow-up period. Although the UK Biobank is a volunteer cohort of generally healthy participants, the cancer incidence rates we observe are similar to those in the general population, consistent with previous findings (36).

This study also has several limitations. First, the study population is restricted to those of “White British” genetic ancestry (UKB Field 22006), as the PRS performs better among individuals of European ancestry (medRxiv 2022.06.16.22276246), and this constitutes almost 95% of the UK Biobank cohort. The numbers of non-white participants were too few for us to assess model performance in women with other ancestries, but previous studies have observed reduced discrimination and poor calibration among Asian and African populations (37, 38).

Second, while calibration of the models was necessary in this analysis to make a fair comparison, we acknowledge that model performance would likely decline in other populations. Some models (e.g., Gail, iCARE) use population-specific baseline rates of breast cancer to adapt for use in multiple populations (31, 39–42), and population-appropriate PRS could also be used in this way, although this requires the availability of relevant population-specific data.

Furthermore, not all variables used in these risk prediction models were available in the UK Biobank. Participants were only asked for a limited first-degree family history of breast cancer; hence we were not able to fully take advantage of the pedigree aspect of the Tyrer-Cuzick model. Information on breast biopsies may be subject to imperfect recall or incomplete ascertainment. Mammographic density, which is known to improve risk prediction in the latest version of the Tyrer–Cuzick model (16, 17, 43, 44), was unavailable. However, mammographic density is not routinely available from medical records in the UK, thus it may not be available for risk prediction in the clinic.

Finally, pre- and postmenopausal breast cancer are known to have different risk factor associations and tumor characteristics, with premenopausal cancers typically being more aggressive and often associated with high-penetrance pathogenic mutations in cancer predisposition genes. Because the UK Biobank population was predominantly (64%) postmenopausal at baseline, we chose to consider only postmenopausal women in our analyses. Additional analyses among premenopausal women are needed, in particular to evaluate the potential benefit of incorporating polygenic risk to prioritize higher risk women for earlier screening.

The costs of running the genome arrays necessary to calculate the PRS are rapidly decreasing and already comparable with many commonly used biochemical tests, and once a contemporary genome array is run multiple PRS for different diseases and traits can be calculated, suggesting that PRS for breast cancer could be routinely available in the future. Our analyses suggest that the addition of a PRS for breast cancer would substantially improve the performance characteristics of these two commonly used breast cancer prediction tools. At a minimum this may provide a means to select participants for primary prevention trials as occurred in the IBIS and STAR trials (45, 46). Further work is needed on whether better breast cancer risk prediction could alter the age at onset or frequency of breast cancer screening sufficiently to justify modification of current guidelines, such as that underway in the WISDOM study (47).

## Supplementary Material

Supplementary Figure S1 Supplementary Figure S1: Analysis population exclusion flowchart. This illustrates the selection of eligible study participants from the UK Biobank.

Supplementary Figure S2 Supplementary Figure S2: Age-specific breast cancer rates in women in the UK Biobank, overall and within analysis cohort, compared to Office for National Statistics (ONS) 2013 Cancer Registry data.

Supplementary Figure S3 Supplementary Figure S3: Schoenfeld residual plots from Cox model containing Tyrer-Cuzick model 10-year risk and PRSBC, in training data (N=101,121).

Supplementary Figure S4 Supplementary Figure S4: Schoenfeld residual plots from Cox model containing Gail model 10-year risk and PRSBC in training data (N=101,121).

Supplementary Figure S5 Supplementary Figure S5: Density plot of 10-year predicted risk with and without PRSBC, split by breast cancer cases and controls in test data (N=25,369).

Supplementary Table S1 Supplementary Table S1: Derivation of variables for the Tyrer-Cuzick model using UK Biobank data.

Supplementary Table S2 Supplementary Table S2: Derivation of variables for the Gail model using UK Biobank data.

Supplementary Table S3 Supplementary Table S3: Descriptive statistics of analysis population by quintiles of breast cancer PRSBC

Supplementary Table S4 Supplementary Table S4: Hazard ratios (HR) associated with variables in the Tyrer-Cuzick model

Supplementary Table S5 Supplementary Table S5: Hazard ratios (HR) associated with variables in the Gail model

Supplementary Table S6 Supplementary Table S6: Reclassification tables for Tyrer-Cuzick model using fixed 5% 10-year risk threshold in test data (N=25,369).

Supplementary Table S7 Supplementary Table S7: Reclassification tables for Gail model using fixed 5% 10-year risk threshold in test data (N=25,369).

Supplementary Table S8 Supplementary Table S8: Model performance using Tyrer-Cuzick v8 approach to Genomics plc PRS inclusion, in test data (N=25,369).

Supplementary Table S9 Supplementary Table S9: Model performance using Mavaddat 313-SNP PRS instead of Genomics plc PRS in test data (N=25,369).
