# Supplementary material for: Assessing the Value of Incorporating a Polygenic Risk Score with Nongenetic Factors for Predicting Breast Cancer Diagnosis in the UK Biobank
Source: Cancer Epidemiol Biomarkers Prev. 2024 Apr 17;33(6):812–20. doi: 10.1158/1055-9965.EPI-23-1432 (PMC11145162; doi:10.1158/1055-9965.EPI-23-1432)
Supplement: Supplementary Figure S1 — Analysis population exclusion flowchart. This illustrates the selection of eligible study participants from the UK Biobank. [file epi-23-1432_supplementary_figure_s1_suppsf1.pdf]

Supplementary Figure S1: Analysis population exclusion flowchart. This illustrates the selection of eligible study participants from the UK Biobank.

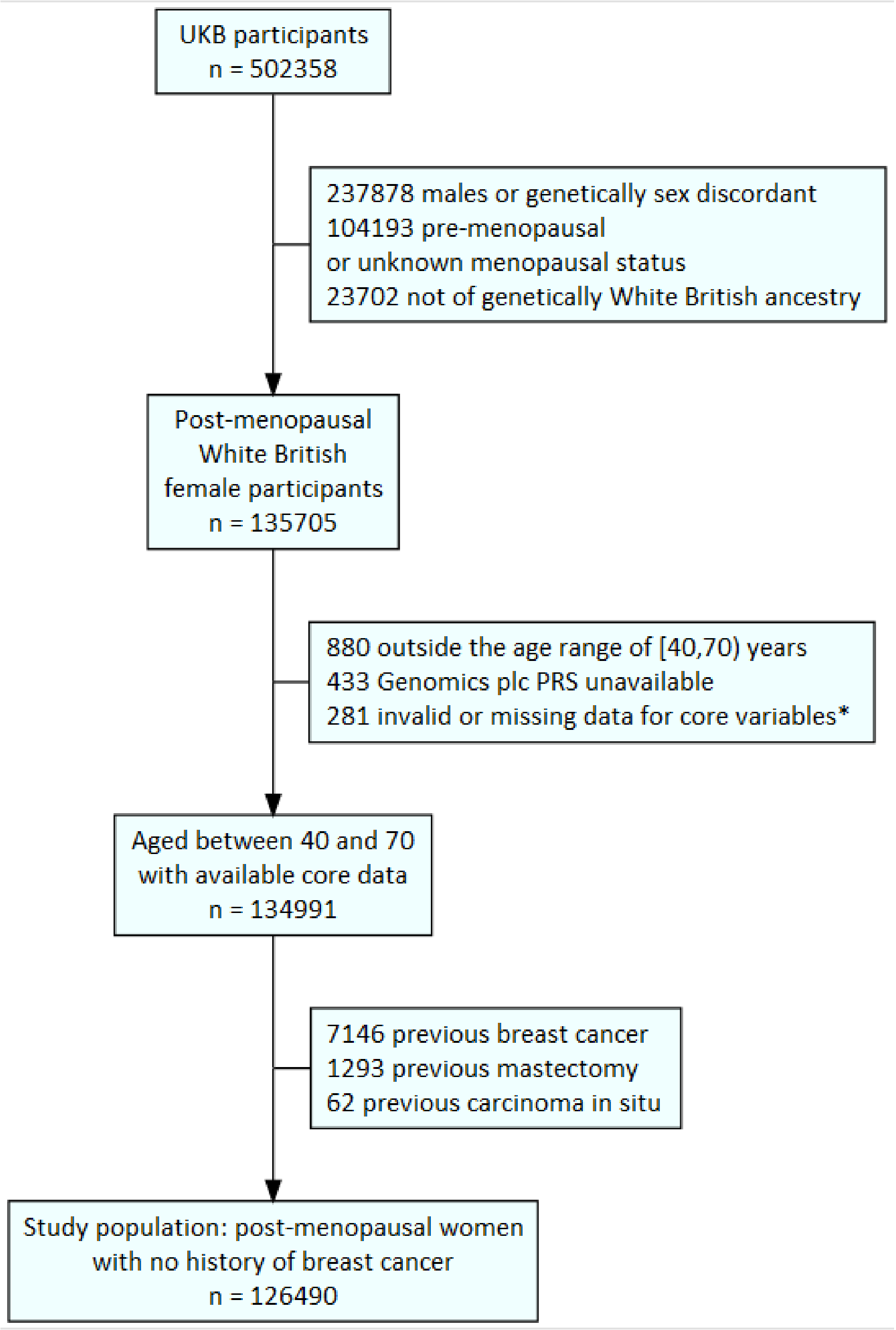

*'Invalid or missing data for core variables' comprises: 1. Missing data for HRT use (Never, Previous or Current) as this variable was required for the Tyrer-Cuzick model. Previous HRT users with unknown age of stopping HRT were categorised into 'Previous (<5)' and 'Previous (>5)' by imputation. 2. Twenty or more sisters. 3. An age of stopping HRT that was greater than age at baseline.
