# Supplementary material for: Assessing the Value of Incorporating a Polygenic Risk Score with Nongenetic Factors for Predicting Breast Cancer Diagnosis in the UK Biobank
Source: Cancer Epidemiol Biomarkers Prev. 2024 Apr 17;33(6):812–20. doi: 10.1158/1055-9965.EPI-23-1432 (PMC11145162; doi:10.1158/1055-9965.EPI-23-1432)

Supplementary Figure S2: Age-specific breast cancer rates in women in the UK Biobank, overall and within analysis cohort, compared to Office for National Statistics (ONS) 2013 Cancer Registry data.

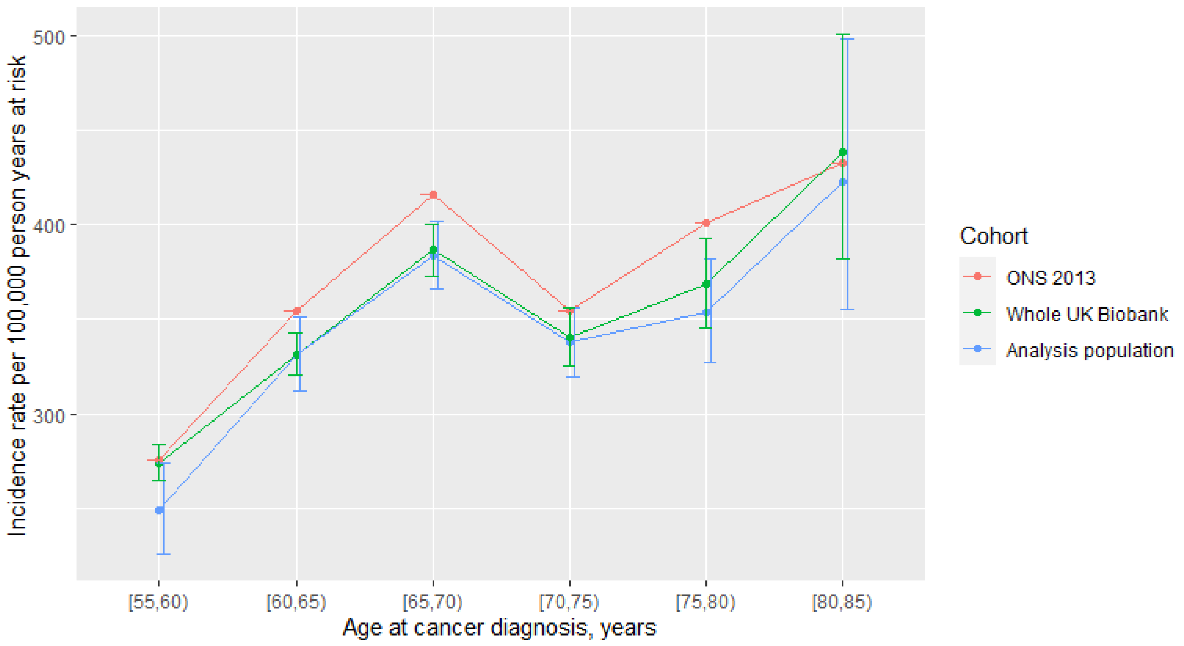

Supplement: Supplementary Figure S2 — Age-specific breast cancer rates in women in the UK Biobank, overall and within analysis cohort, compared to Office for National Statistics (ONS) 2013 Cancer Registry data. [file epi-23-1432_supplementary_figure_s2_suppsf2.pdf]
