# Supplementary material for: Assessing the Value of Incorporating a Polygenic Risk Score with Nongenetic Factors for Predicting Breast Cancer Diagnosis in the UK Biobank
Source: Cancer Epidemiol Biomarkers Prev. 2024 Apr 17;33(6):812–20. doi: 10.1158/1055-9965.EPI-23-1432 (PMC11145162; doi:10.1158/1055-9965.EPI-23-1432)
Supplement: Supplementary Figure S3 — Schoenfeld residual plots from Cox model containing Tyrer-Cuzick model 10-year risk and PRSBC, in training data (N=101,121). [file epi-23-1432_supplementary_figure_s3_suppsf3.pdf]

Supplementary Figure S3: Schoenfeld residual plots from Cox model containing Tyrer-Cuzick model 10-year risk and PRS_{BC}, in training data (N=101,121).

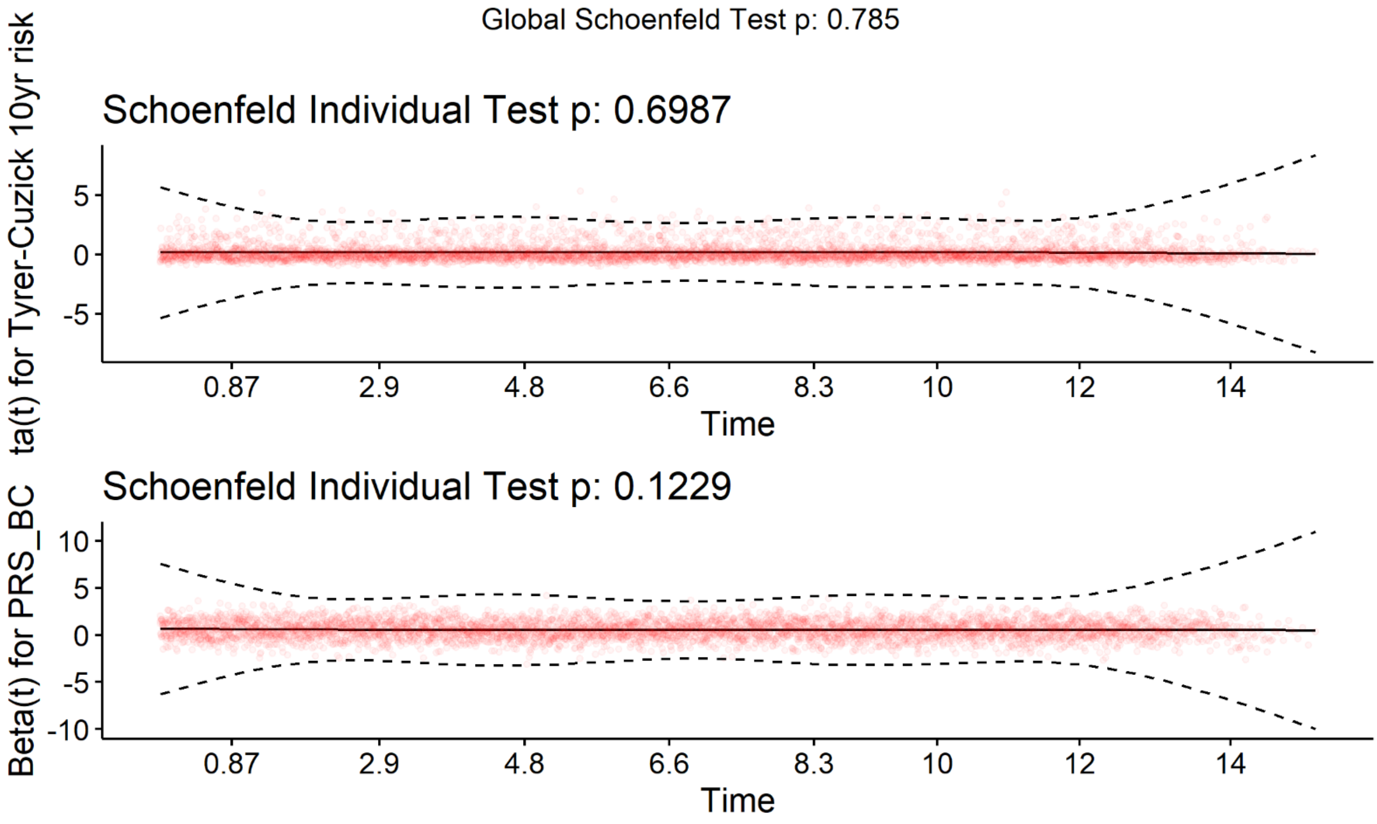

Model also includes genetic array and first 4 principal components of genetic ancestry, plots omitted.
