# Supplementary material for: Assessing the Value of Incorporating a Polygenic Risk Score with Nongenetic Factors for Predicting Breast Cancer Diagnosis in the UK Biobank
Source: Cancer Epidemiol Biomarkers Prev. 2024 Apr 17;33(6):812–20. doi: 10.1158/1055-9965.EPI-23-1432 (PMC11145162; doi:10.1158/1055-9965.EPI-23-1432)

Supplementary Figure S4: Schoenfeld residual plots from Cox model containing Gail model 10-year risk and PRS_{BC} in training data (N=101,121).

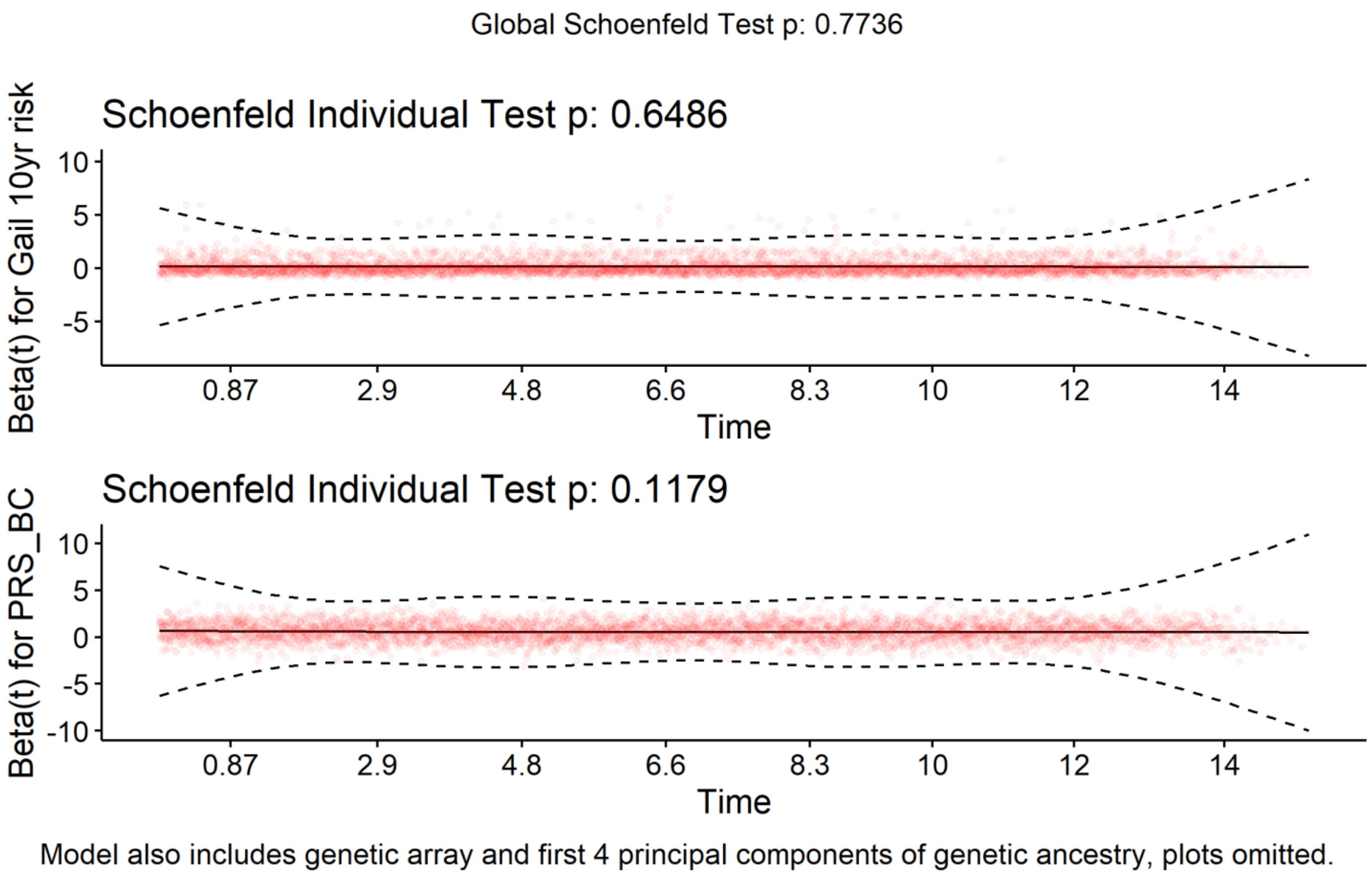

Supplement: Supplementary Figure S4 — Schoenfeld residual plots from Cox model containing Gail model 10-year risk and PRSBC in training data (N=101,121). [file epi-23-1432_supplementary_figure_s4_suppsf4.pdf]
