# Supplementary material for: Assessing the Value of Incorporating a Polygenic Risk Score with Nongenetic Factors for Predicting Breast Cancer Diagnosis in the UK Biobank
Source: Cancer Epidemiol Biomarkers Prev. 2024 Apr 17;33(6):812–20. doi: 10.1158/1055-9965.EPI-23-1432 (PMC11145162; doi:10.1158/1055-9965.EPI-23-1432)

Supplementary Figure S5: Density plot of 10-year predicted risk with and without PRS_{BC}, split by breast cancer cases and controls in test data (N=25,369).

Top panel: Tyrer-Cuzick model, bottom panel: Gail model. The apparent bimodality of the Gail model is caused by first-degree family history of breast cancer.

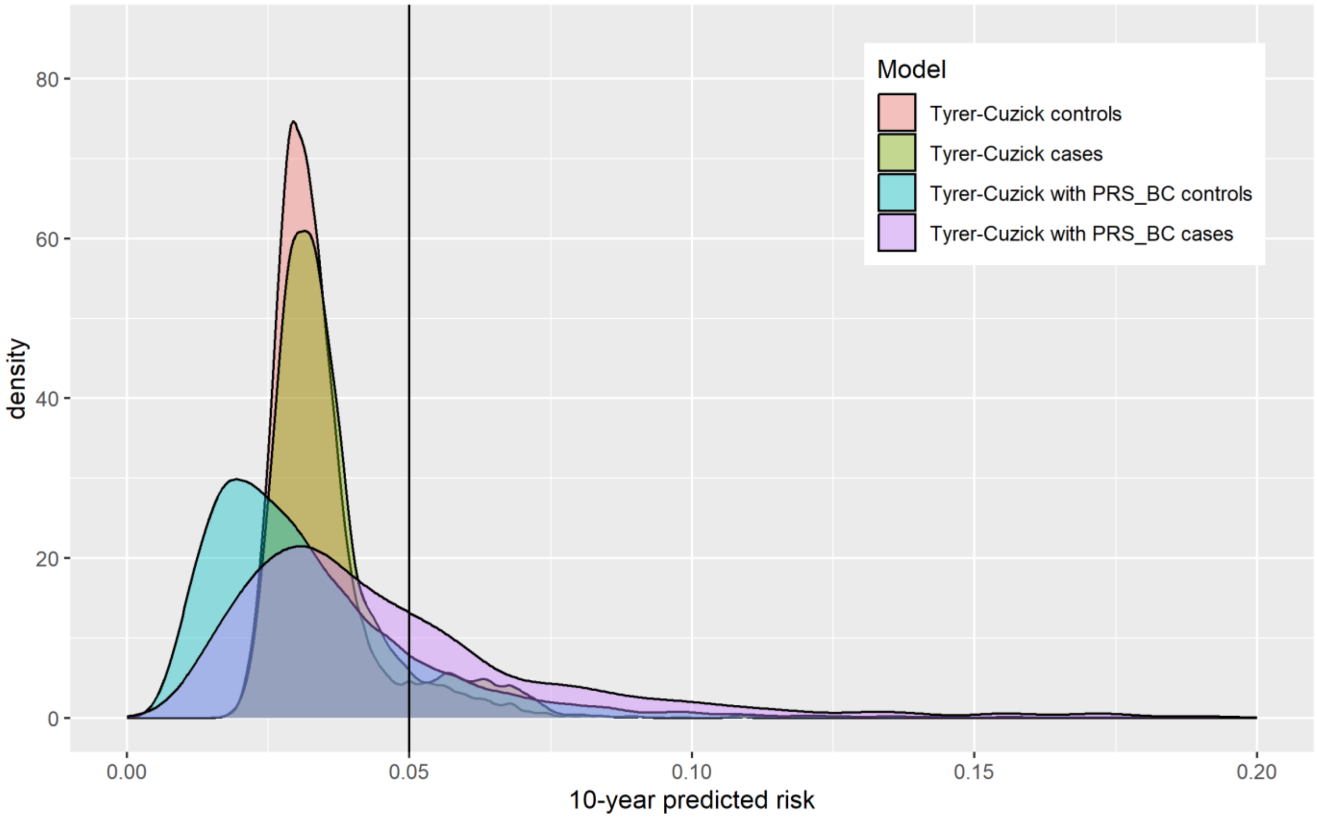

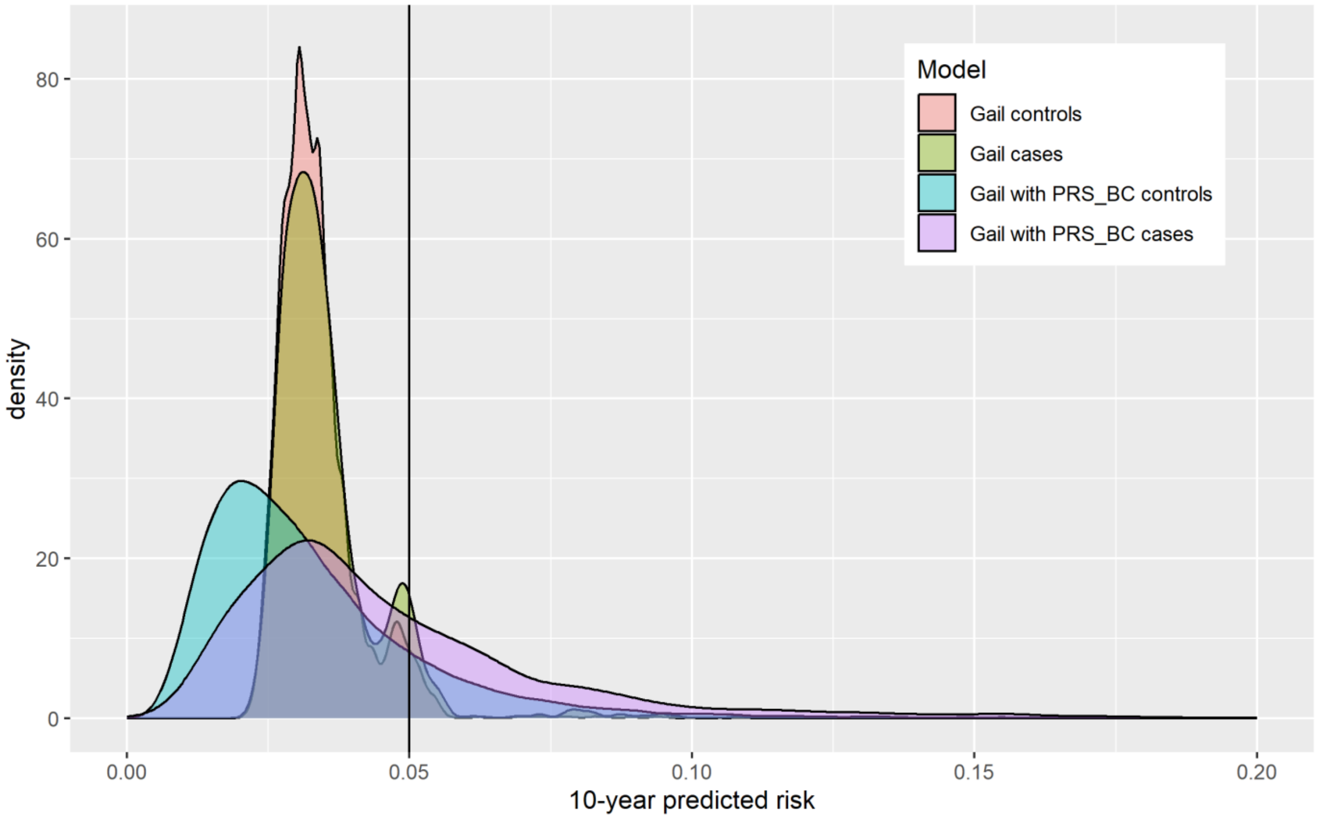

Supplement: Supplementary Figure S5 — Density plot of 10-year predicted risk with and without PRSBC, split by breast cancer cases and controls in test data (N=25,369). [file epi-23-1432_supplementary_figure_s5_suppsf5.pdf]
