# Supplementary material for: Assessing the Value of Incorporating a Polygenic Risk Score with Nongenetic Factors for Predicting Breast Cancer Diagnosis in the UK Biobank
Source: Cancer Epidemiol Biomarkers Prev. 2024 Apr 17;33(6):812–20. doi: 10.1158/1055-9965.EPI-23-1432 (PMC11145162; doi:10.1158/1055-9965.EPI-23-1432)
Supplement: Supplementary Table S1 — Derivation of variables for the Tyrer-Cuzick model using UK Biobank data. [file epi-23-1432_supplementary_table_s1_suppst1.pdf]

Supplementary Table S1: Derivation of variables for the Tyrer-Cuzick model using UK Biobank data.

For the full Tyrer-Cuzick model specification, see https://ems-trials.org/riskevaluator/.

All variables from Tables 1 and 4 of the official Tyrer-Cuzick spec are listed, for Tables 2 and 3 (detailed family history) only variables that could be derived within the UK Biobank are listed.

**Variable description from Tyrer-Cuzick model spec**	**UKB Field IDs used**	**Derivation notes**
Current age (of patient)	52, 34, 53	Estimated date as 15th of the month.
Age at menarche	2714	
Number of live births	2734	
Age at first birth	3872, 2754	Combined data from the field for primiparous women and the field for multiparous women.
Menopausal status	2724	
Age at menopause	3581	
Height	50	
Weight	21002	
History of hyperplasia		Not readily identifiable from ICD codes.
History of atypical hyperplasia	41234	ICD codes used: ICD9 610.8, ICD10 N60.8
History of LCIS		Women with LCIS prior to baseline were excluded for consistency with the Gail model.
History of ovarian cancer	40006, 40013	ICD codes used: ICD9 1830, ICD10 C56*
Age at diagnosis of ovarian cancer	40006, 40013, 40008	
Ashkenazi Jewish heritage		Unavailable for all women.
HRT use	2814, 3546	Women who did not report their HRT use status (Yes/No) were excluded.<br><br>Women who reported previous HRT use without a date of stopping HRT were allocated into the "<5" or ">5 years" categories by imputation.
Type of HRT taken		Not readily available within UK Biobank. While women could self-report the medications they were taking in a verbal interview at baseline.
Length of time taking HRT in the past	2814, 3546, 3536	Women who reported that they had ever used HRT (UKB Data Field 2814) but did not know the age at which they last used HRT (UKB Data Field 3546) were

		regarded as "Previous" users. We further categorised these "Previous" users as whether they stopped HRT <5 years ago (Supplementary Methods).
Length of time woman intends to use HRT in the future (if current user)		Unavailable for all women.
Time since HRT last used (if previous HRT user & time since last use < 5 years)	2814, 3546, 52, 34, 53	
Genetic testing of the woman		Unavailable in the UK Biobank
Has the mother had breast cancer	20110	
Age at which mother developed breast cancer, if not then their current age or the age at which they died	3526	In UK Biobank data, maternal age at breast cancer diagnosis was not available, only age at baseline assessment or age at death.<br><br>Age at maternal breast cancer diagnosis was therefore left blank, maternal age at death was supplied where available.
Number of sisters	1883	
For each sister: has the sister had breast cancer?	20111	We assume that any instance of siblings with breast cancer was a sister unless the participant only had brothers and no sisters, in which case we record a brother with breast cancer.<br><br>Where a participant had multiple sisters, we coded one with breast cancer and the rest without.
Has a brother had breast cancer?		
Has the father had breast cancer?		Unavailable in the UK Biobank
Mammographic density measure		Unavailable in the UK Biobank
Mammographic density value		Unavailable in the UK Biobank
Polygenic SNP score	26220	
