# Supplementary material for: Assessing the Value of Incorporating a Polygenic Risk Score with Nongenetic Factors for Predicting Breast Cancer Diagnosis in the UK Biobank
Source: Cancer Epidemiol Biomarkers Prev. 2024 Apr 17;33(6):812–20. doi: 10.1158/1055-9965.EPI-23-1432 (PMC11145162; doi:10.1158/1055-9965.EPI-23-1432)
Supplement: Supplementary Table S2 — Derivation of variables for the Gail model using UK Biobank data. [file epi-23-1432_supplementary_table_s2_suppst2.pdf]

Supplementary Table S2: Derivation of variables for the Gail model using UK Biobank data.

For the full Gail model specification, see https://bcrisktool.cancer.gov/

Variable in Gail model	UKB Field IDs used	Derivation notes
Current age (of patient)	52, 34, 53	Estimated date as 15th of the month.
Number of benign breast biopsies	41149	OPCS codes used: OPCS3 387, OPCS4 B32
Breast biopsy with atypical hyperplasia	41234	ICD codes used: ICD9 610.8, ICD10 N60.8
Age at menarche	2714	
Age at first birth	3872, 2754	Combined data from the field for primiparous women and the field for multiparous women.
Number of first-degree relatives with breast cancer	20110, 20111	Participants self-reported the breast cancer status of their mothers and siblings, but not of their children.
Ethnicity	21000, 22006	Analyses restricted to women of genetically "White British" ancestry according to UKB Field ID 22006, among which all women were self-reported "White British", categorised as "White".
