# Supplementary material for: Assessing the Value of Incorporating a Polygenic Risk Score with Nongenetic Factors for Predicting Breast Cancer Diagnosis in the UK Biobank
Source: Cancer Epidemiol Biomarkers Prev. 2024 Apr 17;33(6):812–20. doi: 10.1158/1055-9965.EPI-23-1432 (PMC11145162; doi:10.1158/1055-9965.EPI-23-1432)
Supplement: Supplementary Table S3 — Descriptive statistics of analysis population by quintiles of breast cancer PRSBC [file epi-23-1432_supplementary_table_s3_suppst3.pdf]

Supplementary Table S3a: Descriptive statistics of analysis population by quintiles of breast cancer PRS_{BC}

	Q1: lowest (N=25298)	Q2 (N=25298)	Q3 (N=25298)	Q4 (N=25298)	Q5: highest (N=25298)	Total (N=126490)
**Age group, years**						
40-49	715 (2.8%)	688 (2.7%)	666 (2.6%)	661 (2.6%)	650 (2.6%)	3380 (2.7%)
50-59	9475 (37.5%)	9469 (37.4%)	9554 (37.8%)	9596 (37.9%)	9561 (37.8%)	47655 (37.7%)
60-69	15108 (59.7%)	15141 (59.9%)	15078 (59.6%)	15041 (59.5%)	15087 (59.6%)	75455 (59.7%)
**Standing height, cm**						
Mean (SD)	161.94 (6.11)	161.97 (6.10)	162.08 (6.15)	162.13 (6.14)	162.18 (6.13)	162.06 (6.13)
Missing	63	52	59	50	45	269
**Weight, kg**						
Mean (SD)	71.19 (13.67)	70.93 (13.49)	71.03 (13.52)	71.05 (13.44)	71.09 (13.40)	71.05 (13.50)
Missing	81	75	77	73	66	372
**Age at menarche, years**						
Mean (SD)	12.95 (1.58)	12.95 (1.60)	12.95 (1.59)	12.96 (1.60)	12.95 (1.59)	12.95 (1.59)
Missing	697	636	697	678	656	3364
**Parity**						
Missing	14 (0.1%)	18 (0.1%)	14 (0.1%)	8 (0.0%)	12 (0.0%)	66 (0.1%)
Nulliparous	4070 (16.1%)	4000 (15.8%)	4016 (15.9%)	3951 (15.6%)	4004 (15.8%)	20041 (15.8%)
Parous	21214 (83.9%)	21280 (84.1%)	21268 (84.1%)	21339 (84.4%)	21282 (84.1%)	106383 (84.1%)
**Age at menopause, years**						
Mean (SD)	49.69 (5.19)	49.72 (5.11)	49.78 (5.10)	49.84 (5.05)	49.78 (5.16)	49.76 (5.12)
Missing	1673	1671	1636	1624	1560	8164
**HRT use**						
Current	1440 (5.7%)	1472 (5.8%)	1418 (5.6%)	1365 (5.4%)	1395 (5.5%)	7090 (5.6%)
Never	13047 (51.6%)	12977 (51.3%)	13100 (51.8%)	13185 (52.1%)	13189 (52.1%)	65498 (51.8%)
Previous user (less than 5 years ago)	3377 (13.3%)	3461 (13.7%)	3380 (13.4%)	3379 (13.4%)	3292 (13.0%)	16889 (13.4%)

	Q1: lowest (N=25298)	Q2 (N=25298)	Q3 (N=25298)	Q4 (N=25298)	Q5: highest (N=25298)	Total (N=126490)
Previous user (more than 5 years ago)	7434 (29.4%)	7388 (29.2%)	7400 (29.3%)	7369 (29.1%)	7422 (29.3%)	37013 (29.3%)
**Prevalent ovarian cancer**						
No	25236 (99.8%)	25232 (99.7%)	25222 (99.7%)	25243 (99.8%)	25233 (99.7%)	126166 (99.7%)
Yes	62 (0.2%)	66 (0.3%)	76 (0.3%)	55 (0.2%)	65 (0.3%)	324 (0.3%)
**Previous breast biopsy status**						
None	25171 (99.5%)	25145 (99.4%)	25151 (99.4%)	25142 (99.4%)	25122 (99.3%)	125731 (99.4%)
One or more	127 (0.5%)	153 (0.6%)	147 (0.6%)	156 (0.6%)	176 (0.7%)	759 (0.6%)
**Maternal breast cancer status**						
No	23940 (94.6%)	23686 (93.6%)	23485 (92.8%)	23223 (91.8%)	22753 (89.9%)	117087 (92.6%)
Yes	1358 (5.4%)	1612 (6.4%)	1813 (7.2%)	2075 (8.2%)	2545 (10.1%)	9403 (7.4%)
**Number of full sisters**						
Mean (SD)	0.96 (1.13)	0.99 (1.15)	0.99 (1.16)	0.99 (1.15)	0.98 (1.14)	0.98 (1.15)
Missing	307	342	308	345	349	1651
**Sibling(s) breast cancer status**						
No	24558 (97.1%)	24396 (96.4%)	24249 (95.9%)	24110 (95.3%)	23869 (94.4%)	121182 (95.8%)
Yes	740 (2.9%)	902 (3.6%)	1049 (4.1%)	1188 (4.7%)	1429 (5.6%)	5308 (4.2%)

Supplementary Table S3b. Age at first birth among parous women

	Q1: lowest (N=21214)	Q2 (N=21280)	Q3 (N=21268)	Q4 (N=21339)	Q5: highest (N=21282)	Total (N=106383)
**Age at first birth, years**						
Mean (SD)	25.57 (4.85)	25.56 (4.82)	25.58 (4.83)	25.57 (4.82)	25.48 (4.82)	25.55 (4.83)
Missing	12	20	18	22	23	95

Supplementary Table S3c. Duration of HRT use, among women who are currently taking HRT or who previously used HRT less than 5 years ago

	Q1: lowest (N=4817)	Q2 (N=4933)	Q3 (N=4798)	Q4 (N=4744)	Q5: highest (N=4687)	Total (N=23979)
**HRT duration, years**						
Mean (SD)	8.46 (6.05)	8.65 (5.88)	8.55 (5.90)	8.46 (5.93)	8.39 (5.81)	8.50 (5.91)
Missing	501	512	485	470	477	2445

Supplementary Table S3d. Time since stopping HRT among women who had previously used HRT less than 5 years ago

	Q1: lowest (N=3377)	Q2 (N=3461)	Q3 (N=3380)	Q4 (N=3379)	Q5: highest (N=3292)	Total (N=16889)
**Time since last HRT use, years**						
Mean (SD)	2.92 (1.41)	2.89 (1.41)	2.86 (1.43)	2.89 (1.43)	2.86 (1.45)	2.88 (1.43)
Missing	342	333	320	322	331	1648

Supplementary Table S3e. Age at ovarian cancer diagnosis among women diagnosed with ovarian cancer

	Q1: lowest (N=62)	Q2 (N=66)	Q3 (N=76)	Q4 (N=55)	Q5: highest (N=65)	Total (N=324)
**Age at diagnosis of prevalent ovarian cancer, years**						
Mean (SD)	50.94 (11.45)	53.84 (10.46)	53.90 (9.92)	54.49 (9.16)	53.25 (10.18)	53.29 (10.28)

Supplementary Table S3f. Diagnosis of atypical hyperplasia, among women with breast biopsy

	Q1: lowest (N=127)	Q2 (N=153)	Q3 (N=147)	Q4 (N=156)	Q5: highest (N=176)	Total (N=759)
**Atypical hyperplasia**						
No	113 (89.0%)	142 (92.8%)	133 (90.5%)	148 (94.9%)	156 (88.6%)	692 (91.2%)
Yes	14 (11.0%)	11 (7.2%)	14 (9.5%)	8 (5.1%)	20 (11.4%)	67 (8.8%)

Supplementary Table S3g. Maternal age at baseline or death, among women whose mothers had not had breast cancer.

	Q1: lowest (N=23940)	Q2 (N=23686)	Q3 (N=23485)	Q4 (N=23223)	Q5: highest (N=22753)	Total (N=117087)
**Mother's age, at baseline (if alive) or at death, years**						
Mean (SD)	78.22 (11.81)	78.22 (11.69)	78.13 (11.80)	78.07 (11.78)	77.83 (12.04)	78.10 (11.82)
Missing	253	230	282	239	268	1272
