# Supplementary material for: Assessing the Value of Incorporating a Polygenic Risk Score with Nongenetic Factors for Predicting Breast Cancer Diagnosis in the UK Biobank
Source: Cancer Epidemiol Biomarkers Prev. 2024 Apr 17;33(6):812–20. doi: 10.1158/1055-9965.EPI-23-1432 (PMC11145162; doi:10.1158/1055-9965.EPI-23-1432)
Supplement: Supplementary Table S4 — Hazard ratios (HR) associated with variables in the Tyrer-Cuzick model [file epi-23-1432_supplementary_table_s4_suppst4.pdf]

Supplementary Table S4: Hazard ratios (HR) associated with variables in the Tyrer-Cuzick model

Among 92,287 post-menopausal White British women (the training set, restricted to complete case data for these covariates), with 4,022 breast cancer cases.

Tyrer-Cuzick classic risk factors		Adjusted for continuous age		Multivariable*	
		HR	p-val	HR	p-val
**Age at menopause (5y)**		1.08	<0.01	1.08	<0.01
**Age at menarche (y)**	<11	1.05	0.50	1.04	0.61
	11	0.97	0.52	0.97	0.53
	12	1.03	0.52	1.03	0.55
	13 (ref)	1		1	
	14	0.97	0.46	0.97	0.52
	15	0.85	0.01	0.86	0.02
	16	0.98	0.85	1.00	0.98
	17 or older	1.04	0.79	1.06	0.68
**Height (m)**	*<1.6 (ref)*	1		1	
	1.6-1.7	1.17	<0.01	1.17	<0.01
	1.7 or taller	1.34	<0.01	1.35	<0.01
**BMI (kg/m^2)**	*<21 (ref)*	1		1	
	21 to <23	1.05	0.58	1.06	0.51
	23 to <25	1.16	0.06	1.18	0.03
	25 to <27	1.16	0.06	1.20	0.02
	27 or more	1.35	<0.01	1.44	<0.01
**Age at 1st childbirth (y)**	*Nulliparous (ref)*	1		1	
	<17-19	0.91	0.21	0.91	0.21
	20-24	0.89	0.03	0.89	0.03
	25-29	1.02	0.74	1.01	0.81
	30-34	1.09	0.16	1.08	0.19
	35+	1.28	<0.01	1.27	<0.01
**Menopausal hormone therapy**	*Not current (ref)*	1		1	
	Current	1.62	<0.01	1.69	<0.01
**Benign disease**	*None (ref)*	1		1	
	Atypical hyperplasia	1.87	0.21	1.82	0.23

*Multivariable contains all variables in the table, plus age (years)
