# Supplementary material for: Assessing the Value of Incorporating a Polygenic Risk Score with Nongenetic Factors for Predicting Breast Cancer Diagnosis in the UK Biobank
Source: Cancer Epidemiol Biomarkers Prev. 2024 Apr 17;33(6):812–20. doi: 10.1158/1055-9965.EPI-23-1432 (PMC11145162; doi:10.1158/1055-9965.EPI-23-1432)
Supplement: Supplementary Table S5 — Hazard ratios (HR) associated with variables in the Gail model [file epi-23-1432_supplementary_table_s5_suppst5.pdf]

Supplementary Table S5: Hazard ratios (HR) associated with variables in the Gail model

Among 94,210 post-menopausal White British women (the training set, restricted to complete case data for these covariates), with 4,077 breast cancer cases.

**Gail classic risk factors**		**Adjusted for continuous age**		**Multivariable**	
		**HR**	**p-val**	**HR**	**p-val**
**Age at menarche (y)**	<12	1.05	0.22	1.05	0.26
	12 to 13	1.08	0.03	1.07	0.06
	14 or older (ref)	1		1	
**Previous biopsy**	*No (ref)*	1		1	
	Yes	1.46	0.03	1.42	0.04
**Age category**	*<50 yr (ref)*			1	
	>=50yr	1.29	0.02	1.57	<0.01
**Age at 1st childbirth (y)**	*<20 (ref)*	1			
	20-24	1.00	0.94		
	25-29 or nulliparous	1.10	0.11		
	>=30	1.19	0.01		
**Number of first-degree relatives with breast cancer**	*None (ref)*	1			
	1	1.48	<0.01		
	2	2.76	<0.01		
**Interaction: Age at 1st childbirth * first-degree FH**					0.40
<20 (ref)	*0 (ref)*			1	
	1			1.68	
	2			3.15	
20-24	*0 (ref)*			1.05	
	1			1.42	
	2			2.75	
25-29 or nulliparous	*0 (ref)*			1.13	
	1			1.62	
	2			3.28	
>=30	*0 (ref)*			1.16	
	1			2.14	
	2			2.76	

*Multivariable contains all variables in the table, plus age (years). P-value for interaction in multivariable model calculated using the likelihood ratio test.
