# Supplementary material for: Assessing the Value of Incorporating a Polygenic Risk Score with Nongenetic Factors for Predicting Breast Cancer Diagnosis in the UK Biobank
Source: Cancer Epidemiol Biomarkers Prev. 2024 Apr 17;33(6):812–20. doi: 10.1158/1055-9965.EPI-23-1432 (PMC11145162; doi:10.1158/1055-9965.EPI-23-1432)
Supplement: Supplementary Table S6 — Reclassification tables for Tyrer-Cuzick model using fixed 5% 10-year risk threshold in test data (N=25,369). [file epi-23-1432_supplementary_table_s6_suppst6.pdf]

Supplementary Table S6: Reclassification tables for Tyrer-Cuzick model using fixed 5% 10-year risk threshold in test data (N=25,369).

Cases defined as individuals diagnosed with breast cancer within 10 years. Controls defined as individuals who were still at risk of breast cancer by 10 years of follow-up. Individuals censored before 10 years are not displayed.

**Cases**		Tyrer-Cuzick + PRS_{BC}		
		≤5%	>5%	Sum
Tyrer-Cuzick	≤5%	553 (63.06%)	236 (26.91%)	789
	>5%	22 (2.51%)	66 (7.53%)	88
	Sum	575	302	877

**Controls**		Tyrer-Cuzick + PRS_{BC}		
		≤5%	>5%	Sum
Tyrer-Cuzick	≤5%	19150 (82.00%)	2725 (11.67%)	21875
	>5%	707 (3.03%)	773 (3.31%)	1480
	Sum	19857	3498	23355
