# Supplementary material for: Assessing the Value of Incorporating a Polygenic Risk Score with Nongenetic Factors for Predicting Breast Cancer Diagnosis in the UK Biobank
Source: Cancer Epidemiol Biomarkers Prev. 2024 Apr 17;33(6):812–20. doi: 10.1158/1055-9965.EPI-23-1432 (PMC11145162; doi:10.1158/1055-9965.EPI-23-1432)
Supplement: Supplementary Table S7 — Reclassification tables for Gail model using fixed 5% 10-year risk threshold in test data (N=25,369). [file epi-23-1432_supplementary_table_s7_suppst7.pdf]

Supplementary Table S7: Reclassification tables for Gail model using fixed 5% 10-year risk threshold in test data (N=25,369).

Cases defined as individuals diagnosed with breast cancer within 10 years. Controls defined as individuals who were still at risk of breast cancer by 10 years of follow-up. Individuals censored before 10 years are not displayed.

Cases		Gail + PRS_{BC}		
		≤5%	>5%	Sum
Gail	≤5%	564 (64.31%)	272 (31.01%)	836
	>5%	13 (1.48%)	28 (3.19%)	41
	Sum	577	300	877

Controls		Gail + PRS_{BC}		
		≤5 %	>5 %	Sum
Gail	≤5%	19540 (83.67%)	3167 (13.56%)	22707
	>5%	321 (1.37%)	327 (1.40%)	648
	Sum	19861	3494	23355
