# Supplementary material for: Assessing the Value of Incorporating a Polygenic Risk Score with Nongenetic Factors for Predicting Breast Cancer Diagnosis in the UK Biobank
Source: Cancer Epidemiol Biomarkers Prev. 2024 Apr 17;33(6):812–20. doi: 10.1158/1055-9965.EPI-23-1432 (PMC11145162; doi:10.1158/1055-9965.EPI-23-1432)
Supplement: Supplementary Table S8 — Model performance using Tyrer-Cuzick v8 approach to Genomics plc PRS inclusion, in test data (N=25,369). [file epi-23-1432_supplementary_table_s8_suppst8.pdf]

Supplementary Table S8: Model performance using Tyrer-Cuzick v8 approach to Genomics plc PRS inclusion, in test data (N=25,369).

		NRI[a]		
Tyrer-Cuzick Model	Harrell's C (95% CI)	Overall[b]	Case (N=877)	Control (N=23355)
Model only	0.57 (0.55, 0.58)			
Model with PRS_{BC}	0.67 (0.66, 0.69)	0.083 (0.055, 0.108)	0.080 (0.053, 0.104)	0.003 (-0.001, 0.007)
Integrated PRS_{BC}[c]	0.67 (0.66, 0.69)	0.081 (0.052, 0.112)	0.079 (0.050, 0.109)	0.003 (-0.001, 0.006)

[a]NRI: Net Reclassification Index for 10yr risks from model with PRS compared to Calibrated model, with a fixed proportion of women classified as high risk

[b]Overall NRI = Case NRI + Control NRI, where cases are defined as individuals diagnosed with breast cancer within 10 years and controls are defined as individuals who were still at risk of breast cancer by 10 years of follow-up.

[c]Integrated PRS is the output from the Tyrer-Cuzick model run with the PRS as an input, formatted as a log(OR)
