# Supplementary material for: Assessing the Value of Incorporating a Polygenic Risk Score with Nongenetic Factors for Predicting Breast Cancer Diagnosis in the UK Biobank
Source: Cancer Epidemiol Biomarkers Prev. 2024 Apr 17;33(6):812–20. doi: 10.1158/1055-9965.EPI-23-1432 (PMC11145162; doi:10.1158/1055-9965.EPI-23-1432)
Supplement: Supplementary Table S9 — Model performance using Mavaddat 313-SNP PRS instead of Genomics plc PRS in test data (N=25,369). [file epi-23-1432_supplementary_table_s9_suppst9.pdf]

Supplementary Table S9: Model performance using Mavaddat 313-SNP PRS instead of Genomics plc PRS in test data (N=25,369).

		NRI[a]		
Model	Harrell's C (95% CI)	Overall[b]	Case (N=877)	Control (N=23355)
**Tyrer-Cuzick**				
Model only	0.57 (0.55, 0.58)			
Model with PRS_{313}	0.65 (0.63, 0.66)	0.075 (0.053, 0.100)	0.073 (0.050, 0.098)	0.003 (-0.001, 0.006)
Integrated PRS_{313}[c]	0.65 (0.63, 0.66)	0.083 (0.059, 0.107)	0.080 (0.057, 0.104)	0.003 (0.000, 0.006)
**Gail**				
Model only	0.54 (0.52, 0.56)			
Model with PRS_{313}	0.64 (0.63, 0.66)	0.043 (0.023, 0.063)	0.041 (0.022, 0.061)	0.002 (-0.001, 0.004)

[a]NRI: Net Reclassification Index at 10 years of follow-up for 10yr risks from model with PRS_{313} compared to calibrated model only, with a fixed proportion of women classified as high risk

[b]Overall NRI = Case NRI + Control NRI, where cases are defined as individuals diagnosed with breast cancer within 10 years and controls are defined as individuals who were still at risk of breast cancer by 10 years of follow-up.

[c]Integrated PRS_{313} is the output from the Tyrer-Cuzick model run with the PRS_{313} as an input, formatted as a log(OR)
